# Effects of Omeprazole Over Voice Quality in Muscle Tension Dysphonia Patients With Laryngopharyngeal Reflux

**DOI:** 10.5812/ircmj.2292

**Published:** 2012-12-06

**Authors:** Tolga Kandogan, Gökce Aksoy, Abdullah Dalgic

**Affiliations:** 1 Izmir Teaching and Research Hospital, Department of Otolaryngology Head and Neck Surgery, Izmir, Turkey

**Keywords:** Dysphonia, Stomach, Proton Pump Inhibitors

## Abstract

**Backround:**

Laryngopharyngeal reflux (LPR) is the backflow of stomach contents above upper esophageal sphincter, into the pharynx, larynx, and upper aerodigestive system.

**Objectives:**

In this study, effects of omeprazole over voice quality in muscle tension dysphonia with laryngopharyngeal reflux was ınvestigated.

**Patients and Methods:**

Nine patients, 7 males and 2 females, aged between 27-43 (mean age:31) were included to this study. The diagnosis of muscle tension dysphonia with LPR was established by video laryngoscopy, rigid scope 70º. The laryngeal changes related with LPR were evaluated according to Reflux Finding Score. The patients received omeprazole 20 mg twice a day for a period of 6 months. None of the patients received voice therapy. Vocal hygiene guidelines were also explained to the patients. Objective and subjective voice parameters (Jitter, shimmer, NHR, Voice Handicap Index, and Auditive analysis; Roughness, breathiness, and hoarseness) were evaluated in this study.

**Results:**

After treatment with omeprazol, all the parameters showed an improvement in voice quality, but only VHI (P = 0) and shimmer (P = 0,018) are statistically significant.

**Conclusions:**

For FD patients with LPR condition, we highly recommend that LPR treatment should be part of the treatment plan.

## 1. Background

Laryngopharyngeal reflux (LPR) isthe backflow of stomach contents above upper esophageal sphincter, into the pharynx, larynx, and upper aerodigestive system (1). A single reflux episode is assumed also pathological (1). The most common symptom of LPR is hoarseness/dysphonia (92%) (2). Some patients may experience more serious conditions. Less common laryngeal manifestations of LPR include laryngospasm, arytenoid fixation, laryngeal stenosis and carcinoma. LPR is also associated with the development of polypoid degeneration (Reinke's edema), vocal fold nodules and functional voice disorders (3). LPR is also associated with many other head and neck symptoms and diagnoses, can be the sole cause or an etiologic cofactor in the development of many disorders of the aerodigestive tract (3). Muscle tension dysphonia (MTD) is a voice disorder associated with abnormal laryngeal posture or glottic configuration induced by excessive contraction of the laryngeal muscles, and supraglottic contraction is one of the characteristic findings in MTD (4). Dysphonia resulting from increased muscular tension in the larynx and neck is associated with palpably increased phonatory muscle tension in the paralaryngeal and suprahyoid muscles, elevation of the larynx in the neck on increasing vocal pitch, an open posterior glottic chink between the arytenoid cartilages on phonation, and variable degrees of mucosal changes such as vocal nodules or chronic laryngitis 24. Numerous factors may contribute to the development of this disorder, including reflux, stress, and excessive voice use and loudness. Patients with muscle tension dysphonia frequently demonstrate significant emotional stress and manifest other symptoms of muscle tension such as neck and shoulder strain (5).

## 2. Objectives

In this study, effects of omeprazole over voice quality in muscle tension dysphonia with laryngopharyngeal reflux was ınvestigated.

## 3. Materials and Methods

Nine patients, 7 males and 2 females, aged between 27-43 (mean age:31) were included in this study. All of the patients were smokers and the male patients were also social drinkers.The diagnosis ofmuscle tension dysphonia with LPR was established by videolaryngoscopy, rigid scope 70º, (Karl Storz, Germany). The laryngeal changes related with LPRwere evaluated according to Reflux Finding Score (6). The patients received omeprazole 20mg twice a day for a period of 6 months. None of the patients received voice therapy. Vocal hygiene guidelines were also explained to the patients.

### 3.1. Acoustic Analyses

Jitter, shimmer and NHR were analyzed on a sustained [a:] for duration of about 3 seconds using the Multi Dimensional Voice Program (MDVP) with the Computerized Speech Lab CSL 4300B (Kay Elemetrics Ltd., Lincoln Park, NJ, USA). Jitter is a measure of cycle to cycle variability in the period of acoustic signal and detects irregularities in frequency of cycles in the acoustic signal. Shimmer is a measure of cycle to cycle variation in the amplitude of the acoustic signal and a measure of how much intensity of phonation is perturbed from cycle to cycle (7). NHR, noise to harmonic ratio represents a general evaluation of noise present in the analysed signal (8).

### 3.2. Voice Handicap Index

The patients were instructed that these statements are how most people describe their voices and the effects of their voices on their lives. The patients marked the response that indicates how frequently they have the same experience. 0 = Never 1 = Almost Never 2 = Sometimes 3 = Almost Always 4 = Always. 0 to 30 = These are low scores, and indicate that most likely there is a minimal amount of handicap associated with the voice disorder.31 to 60 = Denotes a moderate amount of handicap due to the voice problem. 60 to 120 = These scores represent a significant and serious amount of handicap due to a voice problem (9). The patients completed the VHI scale before and after treatment with PPI.

### 3.3. Auditive Analysis

Roughness, breathiness, and hoarseness were estimated by the attending physician with the patients reading a passage from the Turkishtext “Kasagi“ by Omer Seyfettin, as recommended by Nawka et al. (10). These parameters are estimated as 0 = normal or absent deviance, 1 = slight deviance, 2 = moderate deviance, 3 = severe deviance. Statistical analysis was performed using paired samples t test.

## 4. Results

The pre and post-treatment with omeprazole acoustic data are shown on Table 1. After treatment with omeprazol, all the parameters showed an improvement in voice quality, however, only VHI(P=0) and shimmer(P=0,018) were statistically significant (Table 2).

**Table 1:** Objective and Subjective Evaluation of Voice in the Patients.

Patient	VHI (Pre-post)	RBH (Pre-post)	JITTER (Pre-post), %	SHIMMER (Pre-post), %	NHR (Pre-post), %
1	59-47	(1,1,1)-(1,1,1)	0.39-0.33	5.14-4.21	0.151-0.112
2	61-43	(1,0,1)-(1,0,1)	0.32-0.25	4.43-2.19	0.144-0.231
3	52-41	(1,1,1)-(1,0,1)	0.92-0.97	5.7-5.9	0.171-0.032
4	68-59	(2,1,2)-(1,1,1)	0.51-0.49	6.51-5.87	0.168-0.12
5	63-47	(1,1,1)-(1,1,1)	3.75-2.67	7.27-6.12	0.238-0.231
6	69-53	(2,1,2)-(1,1,1)	1.78-1.44	5.63-4.71	0.143-0.198
7	72-55	(1,1,1)-(1,1,1)	2.05-1.98	3.43-4.19	0.114-0.055
8	57-50	(2,2,2)-(1,2,2)	0.64-0.74	5.89-3.11	0.15-0.119

**Table 2:** The Mean and the Standard Deviations of the Parameters.

	Mean ± SD
**VHI1**	64.33 ± 8.12
**VHI2**	50.22 ± 6.24
**R1**	1.33 ± 0.50
**R2**	1.00 ± 0.00
**B1**	0.89 ±0.78
**B2**	0.67 ± 0.71
**H1**	1.33 ± 0.50
**H2**	1.11 ± 0.33
**JIT1**	1.5008 ± 1.2667
**JIT2**	1.3367 ± 1.0564
**SHIM1**	5.5533 ± 2.8643
**SHIM2**	4.4689 ± 2.4911
**NHR1**	0.17078 ± 4.6997E-02
**NHR2**	0.14500 ± 7.4753E-02

## 5. Discussion

Increased jitter and shimmer have been found to be the acoustic correlates of a voice quality referred to as rough, harsh or hoarse, at least in pathological voices (11). Jitter was found to be one of the best predictors of the severity of both hoarseness and breathiness (12). NHR ratio provides objective information on the presence of breathiness and it is predictive for the severity of both breathiness and roughness (12). Therefore, NHR is believed to be well correlated with the index of hoarseness (13).

Although ambulatory 24-hour double-probe pH monitoring is the gold standard for the diagnosis of LPR, it also can be made on the basis of symptoms and laryngeal findings(14). Within 2 to 3 months of treatment, most patients report significant symptomatic improvement; however it takes 6 months or longer for the laryngeal findingsof LPR to resolve. Thus, twice daily doses of proton-pump inhibitor (PPI) treatment, life style modification and diet restrictions are recommended for a period ofat least 6 months in patients with LPR (8, 14, 15).

LPR incidence in patients with chronic hoarseness is significantly higher than the LPR incidence in healthy individuals (16). LPR had a large, negative influence on the patients voice quality (8). The proper treatment with PPI can significantly reduce the patients' reflux related problems (8, 17). After the treatment with PPI, the typical LPR lesions on the laryngeal mucosa will be diminished to a large extent and the vocal function of the larynx will greatly be improved. Therefore, LPR should not be overlooked in the treatment of dysphonic patients (8). Empiric omeprazole therapy is a reasonable, initial approach to patients with suspected reflux-related posterior laryngitis. A significant number of patients do well with a short course of antireflux therapy (18).

The prevalance of reflux in patients with voice disorders may be as high as 50% (5). Progressively impaired voice quality may be the primary complaint of posterior irritative laryngitis without soreness or other symptoms (7).

There are studies demonstratingdeteriorated voice quality and restricted phonation capabilities in the LPR patients (1, 19, 20).

Oguz et al. reported in their study that the frequency perturbation measures eg. jitter and shimmer were higher in LPR patients (1). Similar results were also obtained by Hanson et al. They studied patients with chronic laryngitis symptoms as they were receiving omeprazole and antireflux precautions. This study showed statistically significantdecreasein jitter, shimmer and improvement in signal to noise parameters after therapy with omeprazole (15). Selby et al. declared that after the patients with LPR received a PPI therapy, a small, but significant improvement was found in the perception of voice quality post-treatment. No significant differences were found between pre- and post-treatment means for any of the acoustic measures except harmonics-to-noise ratio (HNR) (21). Shaw et al. found that omeprazole regimen produced statistically significant improvement in all symptoms except granulomas. In patients with the pretherapy complaint of hoarseness, acoustic measurements of jitter, shimmer, habitual frequency, and frequency range all showed significant improvement.Antireflux therapy with omeprazole is effective, and improvement can be objectively shown (22).

Voice and voice quality are part of a person’s identity and our judgements of others may be influenced by these characteristics. Thus vocal problems can precipitate negative psychological, emotional and social consequences for affected individuals (23). The greatest impact of LPR is likely to be in the area of social functioning, although emotional and psychological well being and role performance might also be significantly affected. Four key symptoms were identified that affected LPR patients: voice problem, chronic cough, throat clearing, and swallowing difficulties, primarly in the context of social and occupational environments.LPR symptoms appear to lead to substantial psychological, emotional and social problems (23). Cesari et al. led to the hypothesis of a possible correlation between duration of the reflux and dysfunction of the arytenoid muscles, upon which chronic vocal fatigue, with consequent laryngeal compensatory stress, depends (24). Belafsky et al. reported similar findings supporting this hypothesis. They stated that MTD may be an indication of underlying glottal insufficiency. In the face of an organic voice disorder hyperkinetic laryngeal behaviors may be used to achieve glottal closure. Such compensatory laryngeal behaviors may mask the correct underlying diagnosis. Abnormal MTP’s are common in patients with underlying glottal insufficiency. Clinicians should be aware that compensatory hyperkinetic laryngeal behaviors may mask an underlying organic condition (25).

Intrinsic laryngeal muscle investigations, especially those of the interarytenoid (IA) muscle, have determined IA muscle anatomy and histochemical and immunohistochemical classification of extrafusal and intrafusal (muscle spindle) fibers. Extrafusal fibers were oxidative type I and glycolytic types IIA and IIX. Intrafusal fibers of muscle spindles were identified by the presence of tonic and neonatal myosin. The results demonstrate that the IA muscle has a phenotype similar to that of limb skeletal muscle. Myosin coexpression, the absence of intrafusal fibers, and fiber type grouping were unusual features found previously in the thyroarytenoid and posterior cricoarytenoid muscles, but they were not present in the IA muscle. These findings lead to the conclusion that the IA muscle has functional significance beyond its assumed importance in maintaining vocal fold position during phonation. The presence of spindles demonstrates differences in motor control as compared to the thyroarytenoid and posterior cricoarytenoid muscles. Further, extrafusal fiber characteristics implicate IA muscle involvement in muscle tension dysphonia and adductor spasmodic dysphonia (26).

Willems-Bloemer et al. reported in their article that patients with LPR related dysphonia showed a significant improvement in their subjective score on dysphonia with the treatment of PPI, lansoprazole 30 mg once daily for 6 weeks (27).Since there are studies confirming that PPI treatment is effective in the treatment of LPR (8, 15), it is not surprising that after treatment of LPR, the voice will be better. Multifactorial etiologies such as LPR are contributing to hoarseness in patients identified with muscle tension dysphonia. An interdisciplinary approach to treating all contributing factors portends the best prognosis (28).

Often patients with FD are also highly likely to suffer from LPR. It is well-known that LPR seriously affects voice quality in patients. LPR's effects on voice quality aremostly caused by posterior laryngitis developed in relation to LPR and by disruptions of mucosal wave consequently. Additionally, LPR may lead to malfunctioning interaritenoid muscles and gaps may develop in this region. Consequently, compensatory muscle spasms will deteriorate laryngeal dynamics and it will lead to further reductions in voice quality.

In our study we observed that PPI treatment lead to improvements in voice quality of LPR patients. Though not a full recovery, patients reported improvements without participation in furhter voice therapies. We believe that in cases of FD with LPR, reductions in voice quality will be felt more strongly. Thus, for FD patients with LPR, we highly recommend that fixing this should be part of the treatment plans. Significant post-treatment reductions in VHI suggests that patients not only benefited from the treatment itselfbut also life-style changes, which is advised in LPR patients.Though not completely statistically significant, it is important to note that we observed improvements in all voice components after treatment with omeprazole. For FD patients with LPR condition, we highly recommend that LPR treatment should be part of the treatment plan.
